# Heteroscedasticity of residual spending after risk equalization: a potential source of selection incentives in health insurance markets with premium regulation

**DOI:** 10.1007/s10198-023-01592-9

**Published:** 2023-05-10

**Authors:** Michel Oskam, Richard C. van Kleef, Rudy Douven

**Affiliations:** 1 Erasmus School of Health Policy & Management, Erasmus Centre for Health Economics Rotterdam, Erasmus University Rotterdam, Rotterdam, The Netherlands; 2 CPB The Netherlands Bureau for Economic Policy Analysis, The Hague, The Netherlands

**Keywords:** Health insurance, Risk equalization, Risk selection, Risk adjustment, I10-health, G22-insurance, Insurance companies, Actuarial studies, H51-Government expenditures and health

## Abstract

Many community-rated health insurance markets include risk equalization (also known as risk adjustment) to mitigate risk selection incentives for competing insurers. Empirical evaluations of risk equalization typically quantify selection incentives through predictable profits and losses net of risk equalization for various groups of consumers (e.g. the healthy versus the chronically ill). The underlying assumption is that absence of predictable profits and losses implies absence of selection incentives. This paper questions this assumption. We show that even when risk equalization perfectly compensates insurers for predictable differences in mean spending between groups, selection incentives are likely to remain. The reason is that the uncertainty about residual spending (i.e., spending net of risk equalization) differs across groups, e.g., the risk of substantial losses is larger for the chronically ill than for the healthy. In a risk-rated market, insurers are likely to charge a higher profit mark-up (to cover uncertainty in residual spending) and a higher safety mark-up (to cover the risk of large losses) to chronically ill than to healthy individuals. When such differentiation is not allowed, insurers face incentives to select in favor of the healthy. Although the exact size of these selection incentives depends on contextual factors, our empirical simulations indicate they can be non-trivial. Our findings suggest that – in addition to the equalization of differences in mean spending between the healthy and the chronically ill – policy measures might be needed to diminish (or compensate insurers for) heteroscedasticity of residual spending across groups.

## Introduction

Contrary to other insurance products, health insurance is often subject to some form of premium regulation to protect affordability of coverage for high-risk people. While car insurers apply the perceived risk of damage to adjust the individual premiums, for instance through age or historical claims data, health insurers are typically obliged to apply community-rating per insurance product. As a result, consumers are charged the same premium for the same product – irrespective of their risk of medical expenditure -, confronting insurers with predictable profits on healthy enrollees and predictable losses on unhealthy enrollees, subsequently resulting in risk selection incentives [22, 32].

To mitigate selection incentives, regulated health insurance markets generally include a system of risk equalization (also known as risk adjustment) to compensate insurers for predictable spending variation using individual characteristics (risk adjusters) such as age, gender, (prior) diagnoses and the (prior) use of specific pharmaceuticals. Through incremental advancements made over the past decades, risk equalization models have become increasingly accurate in compensating for predictable spending variation and reducing predictable profits and losses for subgroups of enrollees.

Studies on the design and evaluation of risk equalization models typically assume (either explicitly or implicitly) that – in the absence of predictable profits and losses—there is no financial incentive for insurers to distort the natural enrollment of individuals. As a result, insurers would need to resort to efficient contracting of care services to generate profit, improving the overall efficiency of healthcare delivery and the functioning of the insurance market [8, 12, 29–32]. Therefore, over the past three decades, research and policy implementations regarding risk equalization design have focused on reducing the predictable profits and losses to diminish the incentives for risk selection (for an overview see [7]). Although the substantial advancements made to risk equalization models did result in notable improvements in predictive strength, the most sophisticated morbidity-based models currently in place do not eliminate predictable profits and losses [18, 30].

Through this paper we make, to the best of our knowledge, a new contribution to the academic literature. We argue that even when risk equalization would perfectly compensate for the predictable profits and losses of identifiable subgroups of individuals, incentives for risk selection remain. In other words, the absence of a predictable profit/loss for risk type *g* does not imply absence of selection incentives towards risk type *g*. Our argument is that the distribution of residual spending (i.e., spending net of risk equalization payments) is likely to vary considerably across risk types. More specifically, the variance of residual spending is likely to increase with expected health spending, exposing insurers to more uncertainty and a greater risk of substantial losses from risk types with high expected spending (e.g., those with a pre-existing condition) than from healthy risk types, ceteris paribus. In the conceptual framework of this paper, we explain and demonstrate how ‘heteroscedasticity’ of residual spending can be a source of risk selection incentives: when insurers are not allowed to risk-rate their premiums, they are likely to prefer risk types with low variance in residual spending over risk types with high variance in residual spending, ceteris paribus. Hence, perfect equalization of the *mean* expected result for different risk types is no guarantee for the absence of selection incentives towards these risk types. Therefore, the primary objective of this paper is to quantify the heteroscedasticity of residual spending. The precise size of the effects will depend on the risk equalization system of an insurance system and corresponding (inter)national regulations. In this paper, therefore, we use the Netherlands as a case study and simulate the selection incentives using Dutch administrative data.

The paper is structured as follows. Sect. "Conceptual framework" provides a conceptual framework on how heteroscedasticity of residual spending may lead to selection incentives using 1) literature on risk management in financial markets, and 2) capital requirements imposed for insurers by regulatory authorities. Sect. "Data and methods" describes the data and methods used for our simulation analysis. In an explorative analysis we compare the standard deviation of residual spending across risk classes to indicate differences in financial uncertainty and compare the 99.5^th^ percentile of residual spending across risk classes to approximate differences in the risk of substantial losses. In Sect. "Results", we simulate the effects of such heteroscedasticity of residual spending on selection incentives. In Sect. "Size of the problem and potential solutions", we discuss the importance of these selection incentives and potential strategies to correct for these incentives. Finally, Sect. "Discussion" summarizes our main findings and their implications.

## Conceptual framework

The problem can be illustrated through the following thought experiment: suppose insurers can select against or in favor of the following two types of consumers: high risks (H) with a pre-existing condition, and low risks (L) without a pre-existing condition. From the viewpoint of insurers, mean per person expected spending equals €1000 for L and €5000 for H. Both types comprise 50% of the population, setting the overall average to €3000. Assume insurers operate in a market with community-rated premiums (per insurance plan) and a sophisticated risk equalization model that perfectly compensates insurers for the difference in *mean* expected spending between L and H. More specifically, insurers receive a risk equalization payment of €2000 (€5000–€3000) for the above-average expected spending of H and contribute a risk equalization payment of €2000 (€1000–€3000) for the below-average spending of L. From the insurers’ perspective, the mean per person expected spending *net of* risk equalization equals €3000 for both risk types (which would be covered by the community-rated premium). So, the insurers’ *expected* financial result for L is equal to that for H. Nevertheless – and that is the key point of this paper – the uncertainty surrounding the expected financial result is likely to be higher for H than for L. The reason is that the standard deviation of the *actual* financial result per individual increases with the level of expected spending, as will be shown later in this paper. Based on two arguments (discussed in Sect. "Variation in uncertainty of financial return" and Sect. "Variation in solvency requirements", respectively), we hypothesize that – under the requirement of community rating – this heteroscedasticity leads insurers to prefer enrollment of L over H, despite the fact that insurers are perfectly compensated for the difference in mean spending between the two risk types.

### Variation in uncertainty of financial return

A first argument why heteroscedasticity of residual spending results in selection incentives stems from the assumption that insurers are likely to prefer a relatively *certain* financial return over a relatively *uncertain* return of the same size. Derived from examples of risk-averse behavior by insurers, such as the uptake of reinsurance or the use of loading factors based on group size, literature suggests that complete risk neutrality of insurers is unlikely [5, 15]. Simplifying the business of insurance, providing coverage of costs for a group of consumers can be considered a financial investment where the insurer invests capital for the operation of insurance and expects a return. Following theory of investment risk, the investor (insurer) is likely to desire a higher *expected* return on his investment if the *actual* return is more uncertain [35]. This implies that – if insurers in our example were allowed to charge risk-rated premiums – they would have been inclined to charge a higher loading fee – or more specifically: a higher *profit mark-up* – to H than to L, ceteris paribus. This assumption corresponds with the work on insurance risk premiums by Kahane [14], stating that the expected return on any investment portfolio incorporates a risk loading fee, proportional to the standard deviation of the portfolio.

Contrary to unregulated markets with risk-rated premiums, regulated markets with community-rated premiums prohibit insurers from charging different profit mark-ups to different groups. In community-rated markets, the variation in profit mark-ups that would have occurred in a risk-rated market serves as an approximation of the selection incentives. Although the exact size of these selection incentives is unknown, metrics from investment risk management can be used to understand the link between the degree of uncertainty and a corresponding desired excess return. When considering multiple stocks for investment, traditional measures to quantify the extent to which one investment option is preferred over another include the coefficient of variation,^1^ the beta coefficient^2^ and the Sharpe ratio [Eq. 1] [3, 24, 25]. While the three measures vary in their specification, they all rely on the standard deviation of (historical) results to quantify the riskiness of investment options. An important beneficial aspect of the Sharpe ratio over the other two measures is its potential to derive the desired excess return on investment for enduring risk. In other words, the Sharpe ratio helps to indicate the desired profit mark-up for specific levels of uncertainty. For investment x, the Sharpe ratio (S) is calculated as follows:

1 $${S}_{x}= \frac{({r}_{x} - {R}_{f})}{\mathrm{Standard \,Deviation }\,({r}_{x})}$$

Here, *r*_*x*_ represents the average return on investment *x* and *R*_*f*_ stands for the best available rate of return of a risk-free asset. For investors deciding between assets for investment, the asset with the largest Sharpe ratio is preferred [24]. Alternatively, by setting a desired Sharpe ratio, the corresponding required value of *r*_*x*_ (= excess return, or ‘price of uncertainty’) can be calculated for any combination of Sharpe ratio and endured standard deviation. Here, the Sharpe ratio reflects a stance of profit-seeking as opposed to the endured risk. We will elaborate on this in the Methods section.

### Variation in solvency requirements

The second argument why heteroscedasticity of residual spending may generate selection incentives lies in the solvency regulations typically instructed to insurers. For example, European Union (EU) legislation requires health insurers to have sufficient financial capital to remain solvent (over the period of one year) with a certainty of 99.5%, or, conversely, to cover a 1-in-200-year catastrophic financial shock [9].^3^. The directive has been implemented to protect insurance firms (and their customers) from financial disasters based on their respective risk profile and capital reserves. Given the difference in variation of residual spending between L and H in our example, the potential financial loss incurred in a 1/200 chance risk of ruin could be significantly higher for groups of H-type consumers than for L-types. Moreover, the magnitude of financial losses above any chosen threshold could be greater for H than for L, ceteris paribus. Consequently, the capital requirements should be higher when enrolling H-type consumers than when enrolling L-type consumers. If insurers were allowed to charge risk-rated premiums, there would be an inclination to charge a higher loading fee – or more specifically: a higher *safety mark-up* – to H than to L, ceteris paribus [23].

Since insurers in our example operate in a regulated market with enforced community-rating (and thus are *not* allowed to risk-rate premiums), the different respective risks of ex-post losses lead insurers to prefer enrollment of L over H. To quantify these incentives, researchers can calculate the difference in solvency requirements when enrolling H versus L. For example: to indicate the risk corresponding to a 1/200 outlier in residual spending, the 99.5th percentile of the distribution of mean residual spending can be calculated for a given portfolio size. By doing this separately for H and L, researchers can approximate the difference in capital requirements for a portfolio of H-type enrollees versus a portfolio of L-type enrollees. Consequently, the ‘cost’ associated with this difference in capital requirements can serve as a proxy for selection incentives under community-rated premiums.

In practice, the EU solvency legislation encompasses more than the 1/200 risk in residual spending. Specifically, in the Commission Delegated Regulation [10],^4^ supplementing the Solvency II Directive [9], the ‘health underwriting risk module’ is introduced as one explicit section of the diversified Solvency Capital Requirement (SCR)^5^ for health insurers. The module covers the premium and reserve risk borne by insurers, dependent of the respective composition of the portfolio of enrollees of an insurer. The contribution to the capital requirement by the health module, from here onwards *SCR*, depends on the following three variables: the total number of enrollees in the portfolio of an insurer, the level of financial reserves, and the annual income through premiums and risk equalization payments. Since the risk equalization payments vary across risk types, the potential enrollment of L versus H, or a group of either type, alters the amount of capital legally required.

Therefore, to comply with the SCR (further operationalized in the Methods section) in a risk-rated market, different safety mark-ups would be applied for the two types of consumers. These theoretical safety mark-ups for the two types may be found through a subsequent analysis on the difference between the respective SCRs and the associated opportunity costs. Moreover, to comply with the solvency requirements, the extra amount of required capital cannot be freely invested elsewhere and will have to be frozen. Potentially, relatively safe investments as government bonds could be allowed but will likely earn the investor fewer returns than unrestricted investments. Alternatively, if an insurer would have to loan the extra capital to comply with the SCR, interest will have to be paid. The cost of capital for the difference in SCR between the two types of consumers therefore serves as a proximation of the selection incentives between these types. Say, for instance, that the per person capital requirements are €400 higher for consumer type H than for consumer type L. An interest rate of 10% on a loan would then imply that the cost of meeting the capital requirement are €40 higher for enrolling an H-type consumer than for enrolling an L-type counterpart. If such costs cannot be reflected in risk-rated safety mark-ups, because of a community-rating mandate, this disparity generates selection incentives for insurers.

### Group size and the law of large numbers

As mentioned above, the group size of individuals pooled is a crucial factor in determining the level of uncertainty borne by an insurer. The law of large numbers reduces both the standard deviation of the mean (residual) spending and the risk of outliers in mean (residual) spending [15]. Hence, the selection incentives that result from both the ‘uncertainty of financial return’ and the ‘risk of ruin’ are also a function of the group size. More specifically, the absolute differences in ‘uncertainty of financial return’ and ‘risk of ruin’ between risk groups are likely to decrease with the size of these groups, as we will discuss and show in our empirical analyses below. This implies that selection incentives might be weaker as insurers can attract larger numbers of individuals from these respected groups.

## Data and methods

The goals of our analyses are 1) to quantify the heteroscedasticity of residual spending across selective subgroups derived from risk adjusters of the equalization model (from here onwards referred to as ‘risk groups’) and 2) to approximate its potential effect on selection incentives. This section describes the data and the methods used to achieve these goals. In order to disentangle ‘heteroscedasticity of residual spending’ and ‘predictable profits and losses’ (i.e., the traditional approach of measuring selection incentives), our analyses are focused on risk groups for which mean residual spending equals zero (which is typically the case for groups that are explicitly flagged by risk adjusters in the risk equalization model, assuming payment weights for these risk adjusters are estimated through the ordinary least squares method).

### Data

The data used for this research comes from the Dutch basic health insurance and was originally used to calibrate the Dutch risk equalization model of 2021. The data include individual-level medical spending covered under the basic health insurance and risk adjuster information for nearly the entire Dutch population in 2018. The risk equalization system includes separate models for somatic care and mental care. In this paper we focus on the former, which covers about 90 percent of total spending under the basic health insurance. The information used to predict individual-level spending contains eleven types of risk adjusters that collectively account for more than 200 risk classes that take the form of dummy variables with a value of 1 (0) for individuals who are (not) a member of that class. The 11 types are as follows: age interacted with gender, institutional status interacted with age, clusters of zip-codes based on regional factors, socioeconomic status interacted with age, household size interacted with age, pharmacy-based cost groups (PCGs), diagnosis-based cost groups (DCGs), durable medical equipment cost groups (DMECGs), multiple-year high cost groups (MYHCs), physiotherapy diagnosis cost groups (PDCGs), and prior spending on home care (PSHC) [27]. For the purpose of this paper, we classify the PCGs, DCGs, DMECGs, MYHCs, PDCGs and PSHC as ‘morbidity adjusters’, as they are directly derived from (prior) utilization of healthcare. In all analyses, we follow the standard procedure of annualizing spending and weighting the outcomes with the fraction of the year the individual was enrolled in health insurance.^6^

### Quantifying the heteroscedasticity of residual spending

To obtain insight into the heteroscedasticity of residual spending from the Dutch risk equalization model, we first replicate the model that was actually in place in 2021.^7^ Next, we identify risk groups defined by risk adjusters, such as age or morbidity status. Since these groups are explicitly flagged by risk indicators in the risk equalization model, mean residual spending of these groups equals zero (a property of the ordinary least squares method that is used here to derive payment weights). In a third step, to quantify the heteroscedasticity of residual spending, we estimate the standard deviation (as a proxy for the uncertainty in residual spending) and the 99.5th percentile of residual spending (as a proxy for the risk of ruin) for each risk group. Variation in these measures across risk groups provides an indication of the heteroscedasticity in residual spending across risk types.

Moreover, given the relevance of group size through the law of the large numbers, we also simulate the standard deviation and 99.5th percentile of the *mean* residual spending for different portfolio sizes. For each risk group *g* and a given portfolio size we run 1000 simulations in which we randomly select *N* consumers from risk group *g* for which we calculate mean residual spending. For these 1000 values we calculate the standard deviation and 99.5th percentile. An increase in portfolio size is expected to result in a decrease of the two measures. We run our simulations for four portfolio sizes: 1000, 10,000, 100,000 and 1,000,000 individuals.

### Quantifying the selection incentives generated by heteroscedasticity of residual spending

The standard deviation and 99.5th percentile of (mean) residual spending do, as standalones, not directly provide an indication of the *size* of selection incentives on a *community-rated market*. To obtain this indication, we simulate the profit and safety mark-ups that insurers would – most likely – have charged to the various types of consumers in a *risk-rated market*.

First, we derive the profit mark-up for risk group *g*: the mark-up that an insurer would charge, based on the uncertainty of the financial return on group *g*, to each individual of that group. As shown in Sect. "Variation in uncertainty of financial return", the desired excess return on an investment, *r*_*x*_, for enduring more uncertainty on its result can be found by selecting a Sharpe ratio, *S*. Typically in equity markets, a Sharpe ratio of 0.2 is found but levels over 2.0 can be achieved for well-returning assets as well as negative ratios for poor performances [1, 13, 34]. Taking a conservative ratio of 0.2 would be conform with typical investment markets. Alternatively, the ratio could be altered to reflect stronger or weaker profit-seeking behavior. It could be argued that not-for-profit semi-institutional insurance markets, such as the basic health insurance market in the Netherlands would gladly accept 0.1 as a ratio, whereas more profit-oriented markets as in the United States may strive for greater ratios (e.g. 0.5).

By converting Eq. 1 (Sect. "Variation in uncertainty of financial return") to Eq. 2, the desired profit mark-up can be found for any number of individuals from a risk group. In this analysis, the return on a risk-free asset, ${R}_{f}$, is set to €0 to simplify comparison. We calculate the mean per person profit mark-up (PMU)*,* for *N* consumers contracted from risk group *g,* as:

2 $${\mathrm{PMU}}_{g,N}= S*\mathrm{Standard \,Deviation} \,({\overline{r} }_{g},N)$$

with *S* the selected Sharpe ratio and the standard deviation of mean residual spending in risk group *g,* and the number of individuals, *N*, selected from that group *g.* The standard deviation of the mean residual spending in risk group *g* for a given *N (*Standard Deviation *(*${\overline{r} }_{g},N$*))* is found through Eq. 3:

3 $$\mathrm{Standard \,Deviation }\,({\overline{r} }_{g},N)=\frac{\mathrm{Standard \,Deviation} \,({r}_{g})}{\sqrt{N}}$$

where the standard deviation of the risk group of individuals (or: individual-level standard deviation) in group *g* (Standard Deviation *(r*_*g*_*))* is divided by the square root of the number of individuals, *N* (i.e., the portfolio size). By doing so, the degree of variation for the number of individuals of the risk group is set to reflect the impact of the law of large numbers which decreases variation. For the analyses it is important to understand the difference between the two metrics of standard deviation.

Combining Eq. 2 and 3 facilitates the calculation of the mean per person profit mark-up for individuals of any risk group. The differences in mean per person profit mark-up across groups indicates the selection incentives under a community-rated premium.

Next, we derive the safety mark-up for group *g*, i.e., the mark-up that insurers would have to charge to fulfill the capital requirements. Assume an insurer *j,* places an insurance plan on the market and, as a consequence, attracts a certain number of individuals from group *g*. EU Solvency II legislation (footnote 4) specifies what capital requirements (SCR_j_) result from that particular chain of events through Eqs. 4 and 5 as follows:

4 $${\mathrm{SCR}}_{j}=3* {\delta }_{j}* {V}_{\mathrm{total},j}$$

Here, ${V}_{\mathrm{total},j}$ is a summed measure^8^ for the volume of reserves of the insurer (${V}_{\mathrm{reserves},j}$) and the annual income through premiums and risk equalization payments (${V}_{\mathrm{incomes},j}$), the latter of which is directly influenced by the composition of the portfolio.^9^ Moreover, ${\delta }_{j}$ reflects a weighted parameter, based on weights set by the EU and the volume measures that make up ${V}_{\mathrm{total},j}$. Equation 5 defines the computation of ${\delta }_{j}$ as follows:

5 $${\delta }_{j}= \frac{\sqrt{{\delta }_{\mathrm{incomes}}^{2} * {V}_{\mathrm{incomes},j}^{2} + {\delta }_{\mathrm{incomes}} * {V}_{\mathrm{incomes},j} * {\delta }_{\mathrm{reserves}} * {V}_{\mathrm{reserves},j} + {\delta }_{\mathrm{reserves}}^{2} * {V}_{\mathrm{reserves},j}^{2}}}{{V}_{\mathrm{incomes},j} + {V}_{\mathrm{reserves},j}}$$

Here, ${\delta }_{\mathrm{incomes}}$ is set to 2.7%, a defined weight by the European Union, specific to the Dutch health insurance market [11].^10^${\delta }_{\mathrm{reserves}}$ is legally set to 5% (footnotes 4 and 10). Note that Eqs. 4 and 5 can be integrated but are rather presented as in the official legislation.^11^ Although there is no parameter for portfolio size, this is taken into account through the total volume of income.

Through Eqs. 4 and 5 we can now find the legally required amount of capital (SCR_j,g,N_) that an insurer would need to hold when *N* individuals from group *g* are contracted. Moreover, by dividing that capital requirement by *N* we find the mean per person capital requirements (CR) for group *g *as follows:

6 $${\mathrm{CR}}_{g}=\left(\frac{{\mathrm{SCR}}_{j,g,N}}{N}\right)$$

Last, to convert the per person amount of capital required for group *g* to a safety mark-up (SMU) for group *g*, the ${\mathrm{CR}}_{g}$ should be multiplied by parameter $\rho$ for the ‘cost of capital’ (e.g., opportunity costs or interest rate on loans) [Eq. 7]. For example, a higher safety mark-up might require the insurer to either maintain a larger sum of own capital resources, effectively reducing the amount of other types of capital, or to attract new capital which results in additional loans. It should be noted that Solvency II for SCR requires that most of the capital to be held by insurers should be own capital, restricting the options for loans or other types of assets. In our analyses, we apply a 5% and 10% rate for $\rho$ to reflect the cost of capital as an indication.^12^ The difference in the resulting safety mark-ups between risk types provides a proximation of selection incentives in community-rated markets, presented by the earlier example of a 10% interest rate on the €400 capital requirements.^13^

7 $${\mathrm{SMU}}_{g}= \rho *{\mathrm{CR}}_{g}$$

### Quantifying the effects of risk sharing on heteroscedasticity of residual spending

In addition to risk equalization, many health insurance markets also include some form of risk sharing (which provides insurers with additional payments based on the actual spending of insured) [2]. One common form of risk sharing is outlier-risk sharing [26], as applied in Germany and the US Marketplaces system [16, 17]. Outlier-risk sharing means that insurers are compensated ex-post by the regulator for (a proportion of) individual-level spending above a predefined threshold. An important motive for such risk sharing is to protect insurers from the risk of large losses [19]. Outlier-risk sharing is likely to mitigate the heteroscedasticity in residual spending. Therefore, as a final step in our analysis, we examine the effect of risk sharing on our measures of interest. More specifically, we simulate the effects of a common form of outlier-risk sharing: 80% cost-based compensation of individual-level spending above a threshold of €100,000.^14^ In line with international standards, the presence of outlier-risk sharing is taken into account in the estimation of the risk equalization model. This means the model is recalibrated on spending *net of* outlier-risk sharing, thereby reducing the total amount of risk equalization payments. As a result, the ex-post compensations are budget-neutral: no extra funds flow into the system. These compensations are, therefore, regarded as incomes to insurers, just as payments from the risk equalization system would be.

## Results

This section presents the results of our analyses. We first report some descriptive statistics of the dataset available for this study (Sect. "Descriptive statistics"). Next, we demonstrate the measures of variance that serve to quantify the heteroscedasticity of residual spending (Sect. "Quantifying the heteroscedasticity of residual spending"). In Sect. "Quantifying selection incentives by simulating group-level profit and safety mark-ups", the two different mark-ups are calculated for a variety of risk groups and in the final part (Sect. "Effects of risk sharing on heteroscedasticity of residual spending"), the effect of risk sharing on our measures of interest is demonstrated.

### Descriptive statistics

Table 1 presents an overview of the various risk groups of individuals chosen for our analyses, representing different levels of prevalence in the population and diverging levels of mean spending. In addition to groups based on age and morbidity adjuster qualification, three other separations have been made. First, diabetics reflect a relatively large group of chronically ill individuals. The cluster of conditions grouped in DCG10 covers a fairly sizeable group (roughly 67,000 individuals) with a substantial level of average spending. Last, the final group of the PCG adjuster is shown to reflect the costliest type of individuals recognized by the risk equalization model: those that use specific and extremely costly pharmaceuticals. For each of these groups mean *residual* spending equals €0 (as a consequence of the ordinary least squares method used to estimate the payment weights of the risk equalization model).^15^ This implies that – despite substantial differences in mean spending across risk groups – there are no predictable profits/losses for these groups, implying the absence of risk selection incentives based on differences in mean residual spending between these groups.

**Table 1:** Descriptive statistics of risk groups

Risk group	Prevalence (%)	Mean actual spending 2018 (€)	Mean predicted spending 2018 by risk equalization (€)	Mean residual spending 2018 net of risk equalization (€)
Entire population	100	2408	2408	0
Individuals aged 0–64	80.7	1600	1600	0
Individuals aged 65 +	19.3	5792	5792	0
No morbidity flags	74.8	1061	1061	0
≥ 1 morbidity flags	25.2	6409	6409	0
Diabetics (PCG12-15)	3.4	8407	8408	– 1
Cluster of conditions (DCG10)	0.4	28,532	28,495	37
Extremely costly pharmaceutical usage (PCG38)	0.0002	602,637	602,638	– 1

*N* = 17,256,295, corresponding to 16,951,700 insured years. Frequencies are calculated as a percentage of total insured years in the population. The risk groups for morbidity flags are derived from grouping all insured that do not qualify for compensation through any of the six morbidity adjusters (‘healthy individuals’) and by taking the complementary group (‘unhealthy individuals’)

### Quantifying the heteroscedasticity of residual spending

**Fig. 1:**
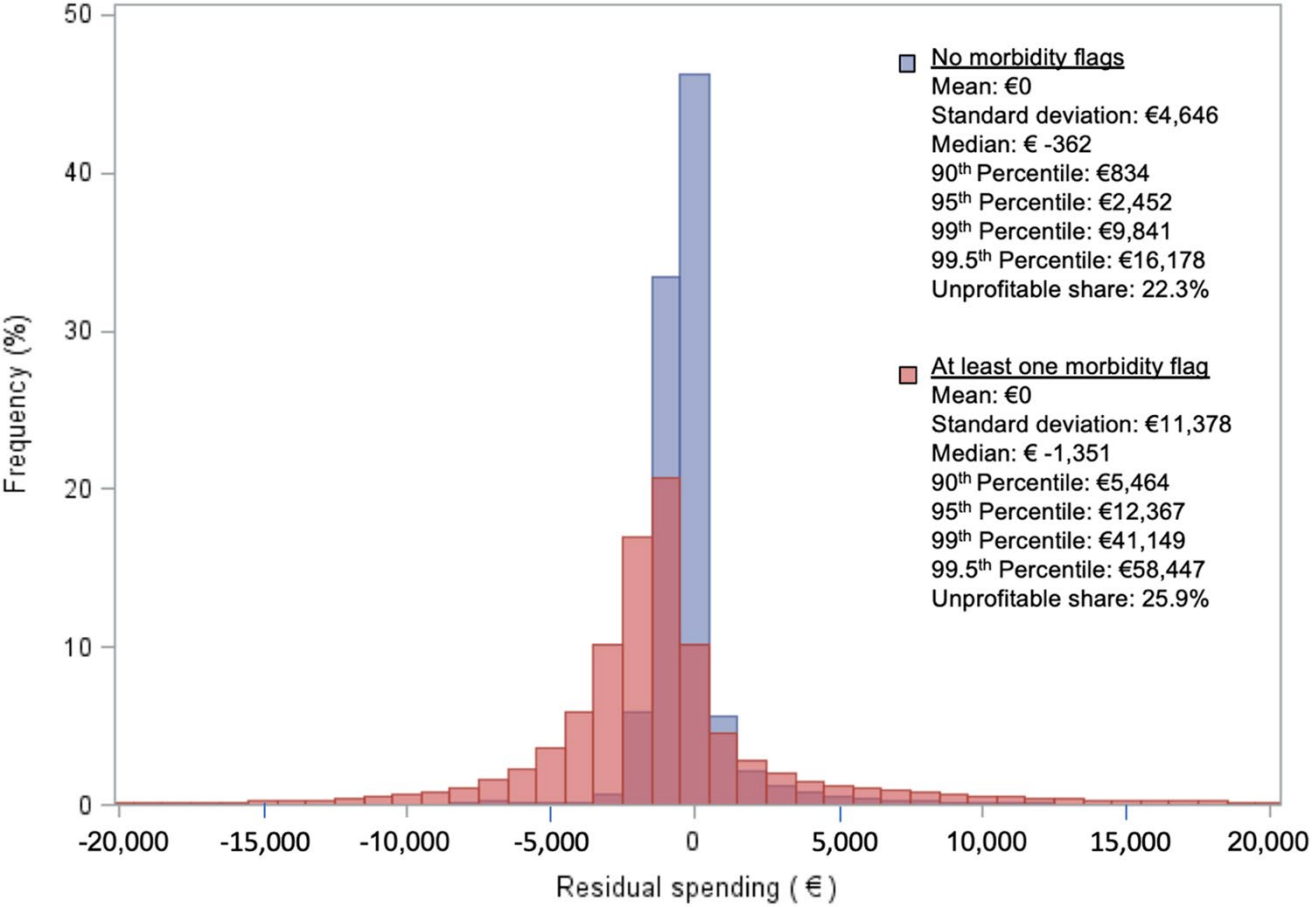
Distribution of individual-level residual spending, separately for individuals without any morbidity flag (*N* = 12,682,389) and those with at least one morbidity flag (*N* = 4,269,311)

To get a first glimpse of the heteroscedasticity of residual spending, Fig. 1 presents the distribution of residual spending for two risk groups as follows: 1) individuals without a morbidity flag (blue) and 2) individuals with at least one morbidity flag (red). Although the graph ranges from €-20,000 to €20,000, the residual spending in the extremes reach well beyond those borders. Nevertheless, roughly 99% of all residuals are captured within the presented range. Note that positive residual spending indicates a loss to insurers, whereas a negative residual represents a profit.

Although the mean residual spending equals €0 for both risk groups, the distributions are quite distinct. In addition to the visual contrast between the two distributions, some metrics are presented to summarize the divergence. The red distribution has a standard deviation of €11,378, far larger than that of the total population (€6978), while the blue distribution has a smaller standard deviation of €4646. The results for the group without morbidity are more concentrated around €0 than those for the group with morbidity, implying that insurers face more uncertainty regarding the ex-post financial result for individuals with a morbidity flag than for those without, despite the fact that the mean residual spending equals zero for both groups. Last, the relative share of individuals that lead to a loss for insurers is greater for the group with a morbidity flag, implying a greater chance of a financial loss when contracting an individual from this group.

**Fig. 2:**
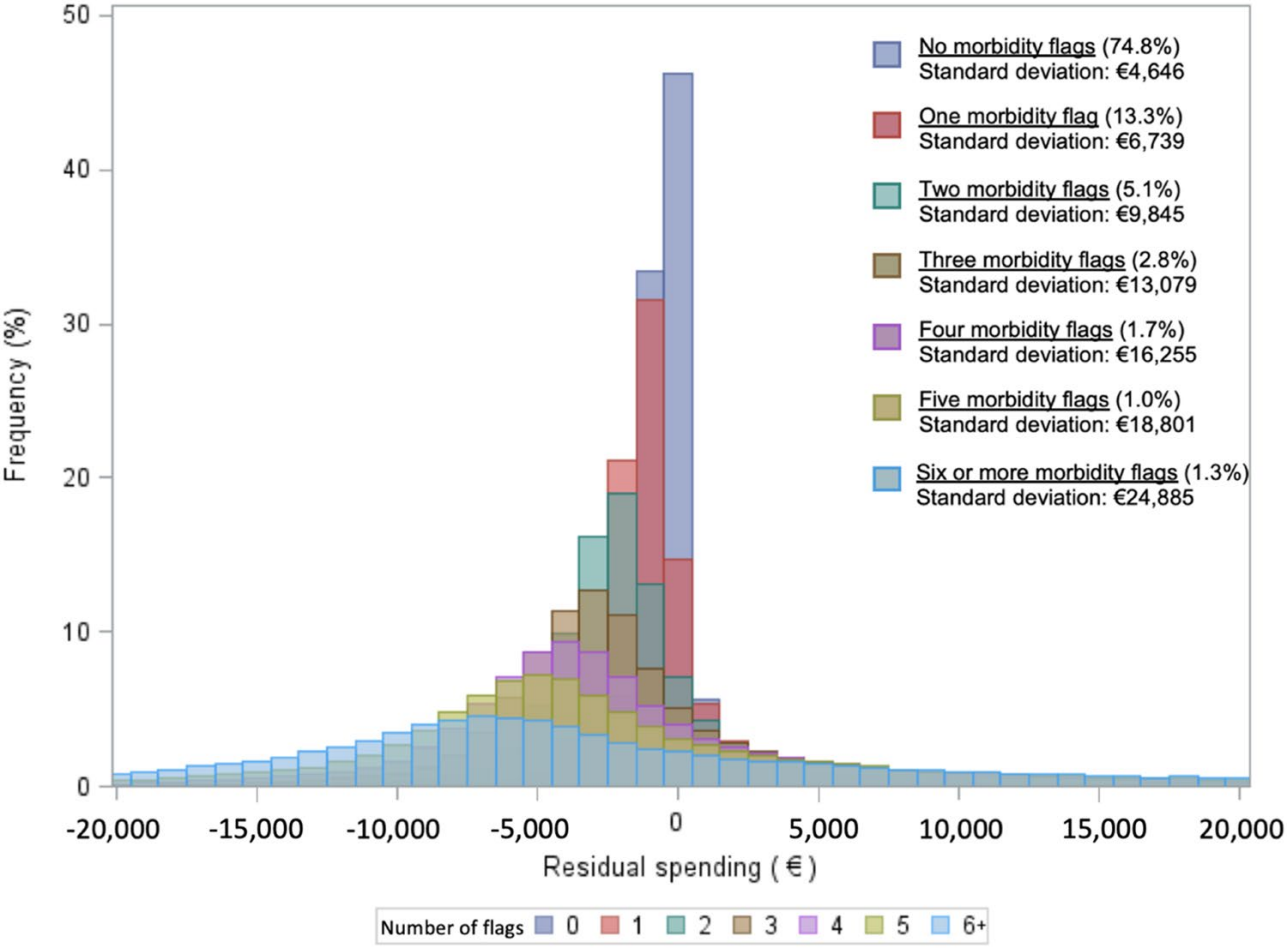
Distribution of individual-level residual spending, separately for groups of individuals based on the number of morbidity flags in the risk equalization model. Note: The mean residual spending of these groups is not exactly €0 (ranging between €- 169 and €49), which is the result of the fact that the number of morbidity flags is not an explicit risk adjuster in the model. Therefore, the average result is not by definition set to €0. Through a small fixed correction (that does not affect the variation in residual spending), the means are set to €0 to concentrate the distributions around the same value for ease of presentation. For instance, if the average result for the group of individuals with precisely two morbidity flags is €49, we lowered the result for all individuals of that particular risk group by a fixed amount of €49

Figure 2 dives deeper into the group with at least one morbidity flag and presents the distributions of residual spending for groups defined by the number of morbidity flags. While the 2021 risk equalization model has six types of morbidity adjusters, consumers can qualify for multiple flags within some of these adjusters, meaning that individuals can end up with more than six morbidity flags. The clear takeaway from Fig. 2 is that the variation of residual spending increases with the number of risk adjustor flags.

**Fig. 3:**
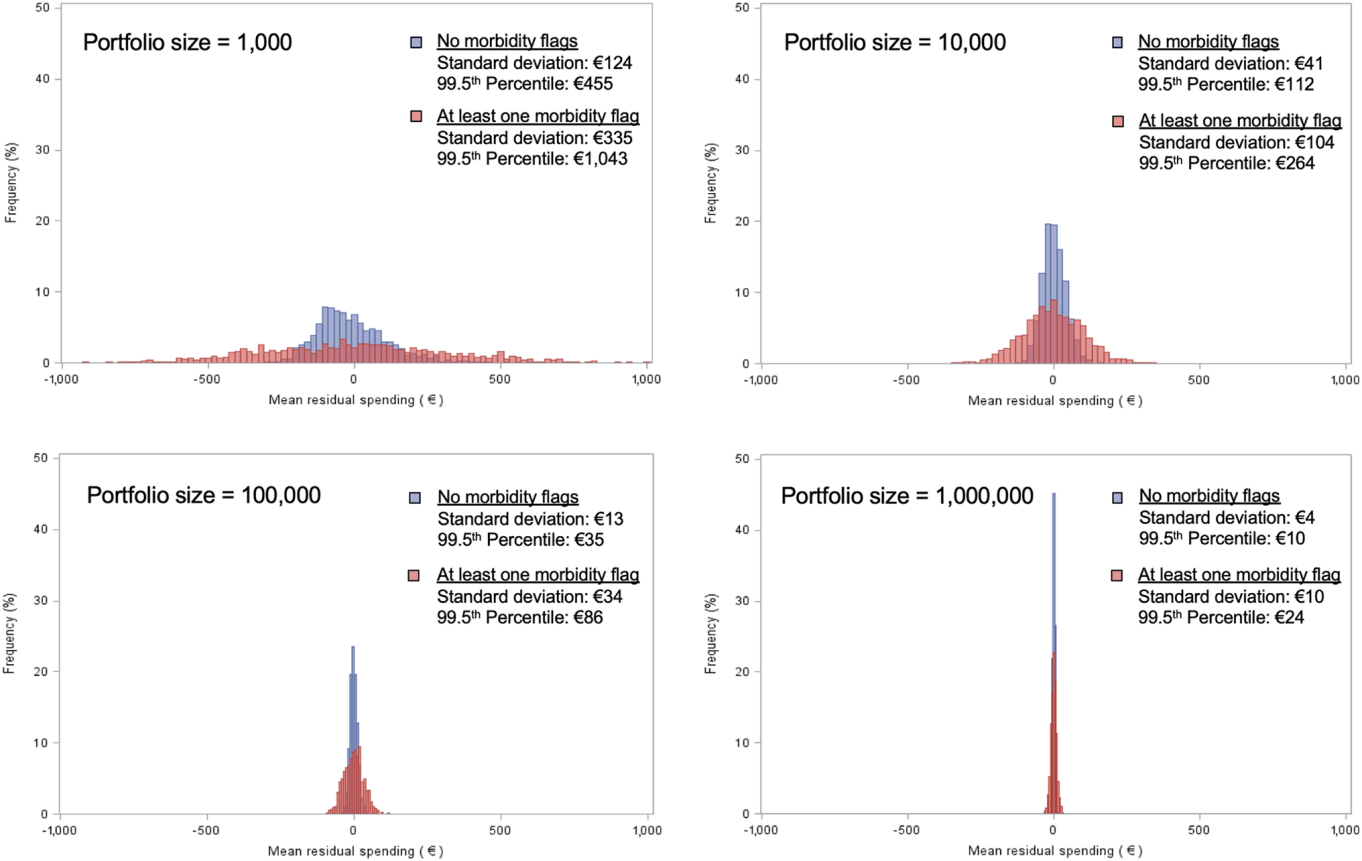
Distribution of mean residual spending for 1000 simulations of portfolio sizes of 1000, 10,000, 100,000 and 1,000,000 consumers, separately for people with at least one morbidity flag and those without a morbidity flag. Note: For each combination of portfolio size and risk group, a thousand random draws with replacement have been performed. The figures show the distribution of mean residual spending for these 1000 draws

While Figs. 1 and 2 present the distributions of residual spending at the level of individual enrollees, Fig. 3 presents the distribution of *mean* residual spending at the level of fictive portfolios, resulting from the simulations. Four portfolio sizes are considered: 1000, 10,000, 100,000 and 1,000,000 enrollees. For every combination of ‘no morbidity flag’ versus ‘at least one morbidity flag’ and the four portfolio sizes, a thousand random draws with replacement have been done to approximate the distribution of *mean* residual spending. In addition to the visual presentation, the variation and 99.5^th^ percentile of mean residual spending decrease with portfolio size, as expected. Taking the portfolio size of 10,000 as an example, the 99.5th percentile is €152 higher for the red group (€264) than for the blue group (€112), while the standard deviation is (€104) is €63 higher for the red group than for the blue one (€41). These values demonstrate the heteroscedasticity of residual spending that remains when the portfolio size is accounted for.

To supplement the results shown in Fig. 3, Table 2 presents the outcomes for the other groups from Table 1. Although all risk groups have a mean residual spending of €0 (considering footnote 15), their distributions of residual spending vary. Both the standard deviation and the 99.5th percentile are notably different for groups with relatively low spending compared to those with relatively high spending. Moreover, the two metrics are derived for portfolio sizes of 10,000 enrollees, similar to the simulations in Fig. 3. The outcomes indicate that insurers face greater uncertainty in the mean residual spending for individuals qualifying for DCG10 (€287) than for ‘healthy’ individuals (€41). The 99.5^th^ percentile of residual spending for the DCG10-group is also considerably higher than for the group without any morbidity flag (€741 versus €126), indicating that insurers would eventually need more financial reserves to endure the 1/200 risk for a portfolio consisting of 10,000 people from DCG10 compared to a portfolio consisting of 10,000 people from the ‘healthy’ group.

**Table 2:** Measures of variance in residual spending for eight risk groups

			Portfolio size (*N*) = 1		Portfolio size (*N*) = 10,000	
Risk group	Total insured years	Mean actual spending (€)	Standard deviation (€)	99.5th percentile (€)	Standard deviation (€)	99.5th percentile (€)
Entire population	16,951,600	2408	6978	33,352	65	191
Individuals aged 0–64	13,685,072	1600	5888	21,371	53	151
Individuals aged 65 +	3,266,628	5792	10,352	55,371	97	247
No morbidity flags	12,682,389	1061	4646	16,178	41	126
≥ 1 morbidity flags	4,269,311	6409	11,378	58,447	106	271
Diabetics (PCG12-15)	538,810	8407	12,234	64,470	123	300
Cluster of conditions (DCG10)	67,345	28,532	29,957	152,530	287	741
Extremely costly pharmaceutical usage (PCG38)	27	602,637	350,078	819,092	*n/a*	*n/a*

For the portfolio size of 10,000, a thousand simulations of random draws with replacement have been performed for the relevant risk group. The standard deviation and 99.5th percentile of residual spending presented are derived from those one-thousand means. The risk groups for morbidity flags are derived from grouping all insured that do not qualify for compensation through any of the six morbidity adjusters (‘healthy individuals’) and by taking the complementary group (‘unhealthy individuals’). The portfolio size of 1 serves to demonstrate the uncertainty/risk when randomly attracting one individual from a specific risk group. The portfolio size of 10,000 serves to demonstrate the uncertainty/risk regarding the mean per person financial result when randomly attracting 10,000 individuals from a specific risk group

For PCG38 it was not meaningful to simulate a portfolio size of 10,000 since – in the total Dutch population – this group consists of no more than 27 individuals. Nevertheless, the existence of such small but very expensive risk groups is highly interesting in the light of the selection problems analyzed in this paper. For bigger groups, insurers have better opportunities to exploit the law of the large numbers by enrolling more individuals. For a group like PCG38 these opportunities are absent: even when insurers would enroll the entire group of people with PCG38, the standard deviation and 99.5th percentile of mean residual spending within the group remain enormous.

**Fig. 4:**
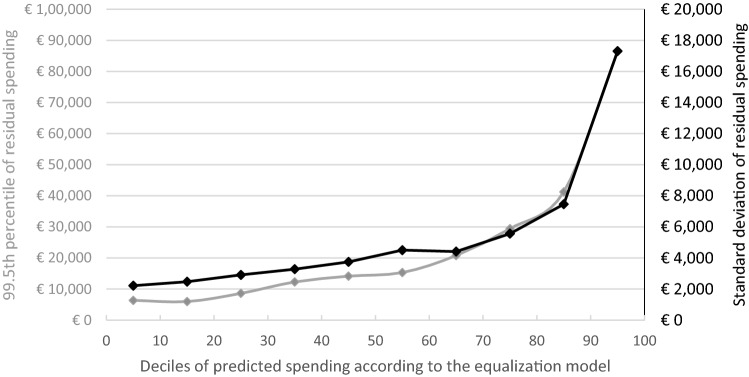
Measures of variance in residual spending (standard deviation in black; 99.5th percentile in light gray) per decile of the population ranked by predicted spending according to the risk equalization model. Note: All insured were ranked by their level of predicted spending according to the risk equalization model. The scales used for the two y-axes are unequal to facilitate comparison in the trends

The results from Table 2 demonstrate an apparent positive correlation between the mean spending of risk groups and the two applied metrics of variation of *residual* spending. To further illustrate this correlation, Fig. 4 shows the standard deviation and 99.5th percentile of residual spending for deciles of individual-level predicted spending according to the risk equalization model. Individuals with low predicted spending are on the left and those with the highest predicted spending are on the right. For the standard deviation and the 99.5th percentile of residual spending a similar pattern is found by using two separate scales on the right and left y-axis, respectively. The clear, non-linear trend observed in the graph demonstrates the substantial concentration of uncertainty in residual spending and risk of large losses among those with the highest predicted spending.

### Quantifying selection incentives by simulating group-level profit and safety mark-ups

To quantify the potential selection incentives that result from the observed heteroscedasticity of residual spending, this section demonstrates the theoretical mark-ups that insurers would have charged to individuals of the different risk groups in a risk-rated market. The motivation for this exercise is that differences in mark-ups among groups provide an indication of selection incentives regarding these groups in a social health insurance market that typically includes community-rating, an acceptance policy for insurers and a defined benefit package. First, the uncertainty of residual spending (standard deviation) is converted to a profit mark-up based on the Sharpe ratio, and second, the safety markup is calculated based on EU solvency legislation and assumptions about the cost of capital.

Table 3 presents the results of the conversion of the standard deviation of the eight respective risk groups to a per contract profit mark-up, derived from specific values of the Sharpe ratio and the number of contracted individuals. The third column shows a hypothetical portfolio of 10,000 individuals from the specific risk groups (except for PCG38, where the risk group includes no more than 27 individuals, shown by the asterisk in Table 3). In the final four columns of the table, two different values of the Sharpe ratio are used for the various portfolios to simulate either a profit mark-up for insurers on a risk-rated market or the selection incentives for insurers on a community-rated market. First, the standard deviation is divided by the square root of the number of enrollees to facilitate the simple application of the Sharpe ratios (Eq. 3). By multiplying the result with a specific Sharpe ratio, the per contract profit mark-up for all of the 10,000 contracts from any risk group can be derived. Moreover, by setting the resulting per capita mark-up for the average individual as a benchmark, a proximation of the selection incentives for insurers towards the individuals from the different risk groups can be simulated.

**Table 3:** Uncertainty of residual spending, the profit mark-up in a risk-rated market and the corresponding selection incentives in a community-rate market

			Portfolio size (*N*) = 10,000			
			Sharpe ratio = 0.1		Sharpe ratio = 0.5	
Risk group	Standard deviation, $\sigma$ (€)	$\frac{\sigma }{\sqrt{\mathrm{10,000}}}$ (€)	Profit mark-up in risk-rated market (€)	Selection incentives in community-rated market (€)	Profit mark-up in risk-rated market (€)	Selection incentives in community-rated market (€)
Entire population	6,978	70	7	–	35	–
Individuals aged 0–64	5,888	59	6	+ 1	29	+ 6
Individuals aged 65 +	10,352	104	10	– 3	52	– 17
No morbidity flags	4,646	46	5	+ 2	23	+ 12
≥ 1 morbidity flags	11,378	114	11	– 4	57	– 22
Diabetics (PCG12-15)	12,234	122	12	– 5	61	– 26
Cluster of conditions (DCG10)	29,957	300	30	– 23	150	– 115
Extremely costly pharmaceutical usage (PCG38)	350,078	3501*	350*	– 343	1750*	– 1715

The PCG38 group only contains 27 insured years; the asterisks (*) for this group indicates that not 10,000 but 27 insured years are contracted. The selection incentives for the risk groups are derived by subtracting the profit mark-up for the respective risk group from the mark-up for the average individual in the population. This simulates a community-rated premium for the overall population and the expected selection incentives for the respective risk groups

Thus, to cover for the difference in uncertainty of financial return between young and elderly individuals, a risk-rating insurer could seek to charge the latter group €10 per contract while requiring €6 per contract from the young, complementary group (assuming a conservative Sharpe ratio of 0.1 for not-for-profit markets). However, taking a less conservative Sharpe ratio of 0.5 and a more uncertain group as DCG10 results in a substantial difference of €127 between that specific group (€150) and the group without a morbidity flag (€23). Notably, the mark-up for the PCG38 group would be substantial, underscoring the severe underlying financial risk for insurers regarding this risk group. Last, by taking the difference between the mark-up for an average individual in the total population (€7 or €35 for a Sharpe ratio of 0.1 or 0.5, respectively) and that for the average individual in risk group *g*, the incentives for risk selection regarding risk group *g* can be simulated for insurers on a community-rated market.

Table 4 provides an indication of how the risk of ruin translates to a safety mark-up. The second column presents the mean predicted spending of the risk groups which reflects the mean per contract income to insurers. This income – which is composed of the annual premium paid by enrollees plus the risk equalization payment received from the regulator – is crucial in determining the safety mark-up (see Sect. "Quantifying the selection incentives generated by heteroscedasticity of residual spending" for further detail). The third column shows the per contract contribution to the solvency capital requirements for an insurer, subject to the assumptions discussed in the Methods section. For instance, a portfolio of diabetes patients results in a capital requirement of €681 per person while a portfolio of individuals without any morbidity flag comes with a capital requirement of €86 per person. Moreover, comparing the age groups results in a difference of €339 between the young (€130) and elderly (€469). Using two values for the parameter for the ‘cost of capital’, $\rho$, we find the respective safety mark-ups. Assuming a cost of capital of 10% relative to the capital requirement, the safety markup for an individual in risk group DCG10 is €211 greater than for an average individual. The differences between most other groups are smaller but non-negligible. Similar to the findings in Table 3, the difference between the respective mark-up for an average insured individual and that for one from a specific risk group provides an indication of the selection incentives for insurers on a community-rated market.

**Table 4:** Risk of ruin and the theoretical safety mark-up for eight risk groups

			$\rho$ = 5%		$\rho$ = 10%	
Risk group	Mean predicted spending (€)	Per *i* contribution to *SCR*_*j*_ (€)	Safety mark-up in risk-rated market (€)	Selection incentives in community-rated market (€)	Safety mark-up in risk-rated market (€)	Selection incentives in community-rated market (€)
Entire population	2408	195	10	–	20	–
Individuals aged 0–64	1600	130	7	+ 3	13	+ 7
Individuals aged 65 +	5792	469	23	– 13	47	– 27
No morbidity flags	1061	86	4	+ 6	9	+ 11
≥ 1 morbidity flags	6409	519	26	– 16	52	– 32
Diabetics (PCG12-15)	8407	681	34	– 24	68	– 48
Cluster of conditions (DCG10)	28,532	2,311	116	– 106	231	– 211
Extremely costly pharmaceutical usage (PCG38)	602,637	48,814	2441	– 2,431	4881	– 4861

The safety mark-up is derived through Eqs 4–7 from the Methods section. The selection incentives for the risk groups are derived by subtracting the safety mark-up for the respective risk group from the mark-up for the average individual in the population. This simulates a community-rated premium for the overall population and the expected selection incentives for the respective risk groups

Although the SCR does not consider any metric of variation, the level of predicted spending is used, which is positively correlated with the variation of residual spending, as previously shown by Fig. 4. Moreover, as discussed before, the SCR hardly considers the portfolio size. Whether the portfolio size equals 1000, 10,000 or 100,000 individuals, the safety mark-ups per contract for those individuals remains equal. The differences between risk groups are therefore retained. These results clash with those presented in Fig. 3, demonstrating that the risk substantially decreases with group size through the law of the large numbers.

### Effects of risk sharing on heteroscedasticity of residual spending

As a final step in our analysis, a risk sharing modality typical for health insurance systems is simulated to concisely demonstrate the impact of such policies on the heteroscedasticity of residual spending. Table 5 demonstrates the effect of outlier-risk sharing – with 80% compensation of individual-level medical spending above a threshold of €100,000 – on the two metrics of heteroscedasticity and the corresponding mark-ups. With most of the costs cut off above the threshold and the risk equalization model recalibrated, the variation in residual spending decreases. Consequently, the profit mark-up decreases too.

**Table 5:** Measures of variance in mean residual spending and the profit and safety mark-ups for two risk groups in a simulation with and without risk sharing supplementing risk equalization

	Std deviation (*N* = 10,000) (€)		PMU (*N* = 10,000) (€)		99.5th percentile (*N* = 10,000) (€)		SMU (*N* = 10,000) (€)	
	Risk sharing		Risk sharing		Risk sharing		Risk sharing	
	No	Yes	No	Yes	No	Yes	No	Yes
No morbidity flag	41	35	5	4	126	92	9	9
≥ 1 morbidity flag	106	96	11	9	271	217	52	52

For the portfolio size of 10,000, a thousand simulations of random draws with replacement have been performed for the relevant risk group. The standard deviation and 99.5th percentile of residual spending presented are derived from those 1000 means. The profit and safety mark-up are calculated as discussed in the Methods section, so the profit mark-up is based on a different standard deviation than shown in the first column. A Sharpe ratio of 0.1 is used in the calculation of the profit mark-up and a cost of capital of 10% for the safety mark-up. The risk sharing modality implies that 80% of costs of enrollees above €100,000 are compensated and the risk equalization model is recalibrated to this adjustment. The values in the final column are equal to demonstrate how the current implementation of the SCR would not incorporate the reduction of risk through risk sharing and that the level of alteration to the inherent parameters that could be considered to do so is unknown

While the 99.5th percentile of residual spending, the risk of ruin, reduces as a consequence of risk sharing, the safety mark-up is unaffected. The fact that the budget is kept neutral in our analysis results in a similar income for the insurers with an average risk profile. Although the overall risk (variation) is reduced through the risk sharing efforts, the SCR does not directly incorporate that effect. The safety mark-up remains equal because the solvency regulations for capital requirements (see Eq. 5) only include mean spending of an insurer population and not the variation in residual spending. Since the risk equalization payments are counted as incomes, the SCR increases accordingly, leading to more capital to be held by insurers. However, if the regulator were to implement a risk sharing modality as a supplement to risk equalization, the parameters in Eqs. 4 and 5 can be altered by the governing body to reflect the decreased degree of risk borne by insurers. Nevertheless, in line with the expectations, the risk sharing modality commonly applied in health insurance markets reduces heteroscedasticity of residual spending.

## Size of the problem and potential solutions

In this section we discuss the extent to which the selection incentives caused by heteroscedasticity of residual spending are problematic. The extent of the problem depends on two factors: 1) the size of the incentives, and 2) the options for insurers to engage in risk selection. Additionally, we explain various strategies that could be considered to mitigate the selection incentives due to heteroscedasticity of residual spending.

### The extent of the problem of risk selection incentives

Through our simulations, we illustrated how heteroscedasticity of residual spending after risk equalization confronts insurers with selection incentives. Using Dutch health insurance data of the total population we find considerable differences in the standard deviations of residual spending between different risk groups of insured despite the fact that the mean residual spending equals €0 for the considered groups. We simulated how – in a risk-rated market – this heteroscedasticity would most likely have resulted in different profit and safety markups. For example, for two mutually exclusive groups based on morbidity status – i.e., individuals without any morbidity flag in the equalization model versus those with at least one morbidity flag—we found a difference in profit mark-ups of €6 for a conservative Sharpe ratio of 0.1 and €34 for a less-conservative Sharpe ratio of 0.5. These findings are, however, subject to assumptions on portfolio size: smaller sample sizes increase uncertainty and thereby mark-ups. Insurance markets with larger portfolios will therefore endure less concerns of uncertainty. Nevertheless, for specific risk groups of individuals with few overall members (such as PCG38 in our analyses) uncertainty will always be large. Moreover, our simulation of the EU solvency legislation indicates a difference in safety mark-ups between the groups with and without at least one morbidity flag of €22 with the ‘cost of capital’ parameter set to 5% and €43 when that parameter is doubled to 10%. Taking the profit and safety mark-ups together creates a per capita difference of €77 (€34 profit mark-up and €43 safety mark-up) between the groups with and without at least one morbidity flag. These findings imply that on a community-rated insurance market (where insurers must charge the same premium to these groups), contracting an individual from the non-morbidity group is €77 more appealing to insurers than contracting one from the complementary group of individuals with at least one morbidity flag. Clearly, greater differences can be found when comparing ‘healthy’ individuals with specific risk groups for which the variation in residual spending is (much) larger than for the total group with a morbidity flag. The size of these selection incentives, however, strongly depends on the parameters and assumptions underlying our analyses. In general, the selection incentives (as quantified above) will be greater (smaller) for markets with higher (lower) demands for insurance firm profit-return or stricter (looser) solvability requirements by regulators. As for the Dutch case, however, the simulated selection incentives that result from heteroscedasticity of residual spending can be considered non-trivial.

However, the fundamental question remains to what extent the incentives evolve into actions and thus become problematic. Although direct rejection of prospective enrollees is prohibited in many health insurance systems (due to open enrollment requirements), a variety of actions of risk selection can be legally undertaken to distort the natural enrollment of individuals [4, 28]. One example of risk selection is insurers not contracting the best care for enrollees with a (specific) chronic disease that are known to be unprofitable. Other examples include offering high premium discounts for the uptake of a voluntary deductible, the design of supplementary insurance plans, and selective marketing towards groups that are known to be profitable [4].

Selection activities are often subtle, and providing clear evidence of risk selection is not straightforward [33]. One way to demonstrate risk selection is to compare insurer-level residual spending net of risk equalization payments for two consecutive years. The glaring problem, however, is that any differences might also be related to other factors, such as efficiency. Nevertheless, *signals* of risk selection, as the forms discussed above, are prevalent and provide an indication of insurers exploiting the knowledge on predictable profitability of risk groups. For example, such signals include the offering of so-called ‘twin products’ and the targeting of specific groups (such as highly-educated people).^16^ Other signals include complaints by insurers that the predictable losses on chronically ill people discourage them to organize the best care for chronically ill people [28].

### Solutions to the problems that result from heteroscedasticity of residual spending

If the heteroscedasticity found in this study is considered problematic for the functioning of the insurance market, the current focus of risk equalization design (i.e., compensating for differences in mean spending across risk types) is insufficient. Even if the risk equalization model would perfectly compensate insurers for differences in mean spending across risk types, heteroscedasticity in residual spending remains. In general, we see three potential solutions to mitigate the selection incentives that result from heteroscedasticity in residual spending.

A first solution may be to supplement risk equalization with risk sharing. In this study, we simulated how a common form of risk sharing, outlier-risk sharing, reduces heteroscedasticity of residual spending. These findings imply that risk sharing indeed provides the regulator with an instrument to mitigate the type of selection incentives studied in this paper. However, risk sharing also comes with a price: incentives for insurers to control costs are reduced. Recent studies on innovative forms of risk sharing demonstrate how the tradeoff between selection and efficiency can be mitigated: targeting the ex-post compensations to individuals with high residual spending instead of high spending [17, 18]. Moreover, ex-post compensations for high residual spending can be financed by ex-post repayments for extremely low residual spending. Targeting the extremes in residual spending cuts off the wide edges of the distribution of residual spending, reducing heteroscedasticity. Alternatively, risk sharing could be applied conditionally on specific risk groups, e.g., those groups with the highest variance in residual spending.

The second solution could be to modify the risk equalization payments and compensate insurers ex-ante for the heteroscedasticity in residual spending. For example, the regulator could estimate the selection incentives due to heteroscedasticity in residual spending (following the methods of this paper) and modify the risk equalization payments for risk groups to eliminate these incentives. In general terms, this means more (less) compensation for risk groups with a relatively high (low) variance in residual spending. Returning to the findings of our simulations, the difference in mark-ups between risk groups could be used as a basis for modifying the risk equalization payments. Taking the results in Table 4 as an example, the equalization payments for individuals without a morbidity flag could be decreased by €11, while the payments for the complementary group with morbidity flags could be increased by €32. While this strategy would render risk groups that are potentially vulnerable to actions of risk selection more financially appealing to insurers, the design – like our research – involves assumptions with respect to Sharpe ratio and financial reserves of insurers. To some extent these assumptions will be surrounded with uncertainty which creates the potential of under/overshooting. Further research is required to determine the optimal compensations to adequately alleviate the risk selection incentives from heteroscedasticity.

A third solution could be to permit limited differentiation in mark-ups in premium setting across individuals, diminishing the risk selection incentives at the cost of health insurance affordability and solidarity. With perfect equalization – as assumed in this study – the mark-up will reveal the different profit and safety mark-ups that insurers require for different risk types. The advantage of this approach over the second solution (as discussed in the previous paragraph) is that premium differentiation does not require any (arbitrary) assumptions to be made by the regulator about hypothetical profit and safety mark-ups. However, existing risk equalization models do not yet perfectly compensate for differences in mean spending across risk types. Allowing risk-rated premiums could lead insurers to not only reflect differences in profit and safety mark-ups across groups but also differences in predictable profits and losses across these groups. This could exacerbate the negative impact on affordability and fairness.

All in all, we expect the uncertainty of the financial result on a group-level to result in selection incentives for competing insurers, even when the risk groups are correctly compensated for their mean expenditure. The actions that insurers in regulated markets may undertake to evade the enrollment of individuals from ‘risky’ groups are problematic to the functioning of the insurance system. In the hypothetical scenario with perfect risk equalization on a group-level, various solutions are possible to decrease the heteroscedasticity in residual spending and the resulting selection incentives but all come with respective challenges and tradeoffs. In the next, and final, section we reflect on the implications of our research as a whole and to what extent intervention is required.

## Discussion

Our conclusion that residual spending in Dutch basic health insurance is subject to substantial heteroscedasticity follows directly from the data. However, the subsequent quantification of selection incentives in a community-rated market (through simulation of the profit and safety mark-up in a free market) incorporates assumptions. The profit mark-up is built upon the Sharpe ratio, a metric used in investment risk management and not specifically designed for health insurance markets with mostly not-for-profit insurers, as in the Netherlands. Despite the use of a conservative value for the Sharpe ratio in our study, it should be further explored how this metric relates to the actual risk health insurers face in practice. In for-profit markets the Sharpe ratio of competing insurers, or any other approximation of profit-seeking behavior, is likely to be higher than in non-profit markets. More generally, the strength of the incentives resulting from heteroscedasticity does not only depend on the size of the loadings that we identify but also on the behavior of the insurers and how they respond to financial incentives. Obviously, the interpretation of the results is subject to such uncertainty.

The calculations of the safety mark-up depend upon a baseline level of financial reserves (i.e. the financial reserves insurers already possess before the start of a new contract period), or rather the absence thereof. Additional analyses show that the magnitude of the safety mark-up, based on the solvency capital requirements, remains relatively similar when using different starting levels of financial reserves (Table 6 in the Appendix demonstrates the effect of different levels of financial reserves on the resulting safety mark-ups for our considered risk groups). Remarkably, an *increase* of the baseline level of financial reserves results in an *increase* of the mark-up (or the contribution to the capital requirement), and the increase of the mark-up is nearly equal between the various risk groups analyzed, despite the differences in ‘risk’ (average expenditure of the group). The overall increase is more or less linear, resulting from the increase to financial reserves alone and almost entirely irrespective of the ‘risk’ of the respective risk groups. In practice, however, the initial level of financial reserves level may play a more crucial role. A substantial share of the mandatory capital for the SCR is required to be an insurer’s own, while in our analysis we used only the ‘cost of capital’ to quantify the selection incentives. However, insurers with insufficient financial reserves may have to charge an additional fee to raise their own resources in addition to the costs of loans or alternative opportunity costs. As a result, insurers with different levels of financial reserves might have to charge different premiums to the same individual. Building up sufficient capital resources through higher premiums would therefore be an alternative, although such premium rises might deter potential enrollees.

A limitation of our study is that we focused on ‘random risk’ assuming the absence of ‘macro risk’. More specifically, we considered the risk for an insurer to randomly attract high residual spenders from a risk group that – on average – has mean residual spending of zero. In practice, however, insurers are also subject to macro risk (i.e., the risk that – at the population-level – the mean residual spending for risk group *g* exceeds or falls below zero), e.g., due to inflation, a pandemic or unforeseen price deviations concentrated in specific risk groups. For instance, pharmaceutical or technological advances in specific disease areas may disrupt the overall financial macro risk faced by the insurers. Such deviations at the macro scale might be more prevalent and severe for ‘riskier’ groups of individuals (i.e., individuals of risk groups with high average health expenditure). Therefore, the selection incentives due to heteroscedasticity of residual spending found in our research may be an underestimation of the level of these type of selection incentives in practice. More research is needed to extend our analysis of random risk with elements of macro risk.

Through another perspective, it could be argued that more variance in residual spending may indicate larger potential gains for insurers through efficiency, as it presents insurers more room to contract and provide healthcare efficiently. However, this argument is subject to the degree of efficiency already present in the insurance market itself. Additionally, such potential endeavors by insurers are again subject to risk aversion since the focus on a particular disease type may still attract unfavorable individuals from the costlier end of the particular risk group. Moreover, the fear for potential ‘bad draws’ may offset the appeal for the large potential gains to be made from a volatile group such as FCG38.

For the purpose of this research, we have assumed a risk equalization model that perfectly compensates for differences in mean spending across risk types. In practice, however, risk equalization models do not fully compensate for these differences in mean spending. One specific option for improvement of the Dutch model could be the implementation of additional morbidity indicators (e.g., diagnostic cost groups based on ICD-10, once this information is available for the entire Dutch population). The central point of our paper, however, is that even when the risk equalization model would perfectly compensate for differences in mean spending across risk types, some selection incentives may remain due to the substantial heteroscedasticity in residual spending. Consideration of this heteroscedasticity and its implications is therefore essential in the process of shaping and evaluating risk equalization and risk sharing systems in health insurance markets.

## Data Availability

The used data are strictly confidential and requires a signed agreement of permission by the Dutch Ministry of Health, Welfare and Sports and the Central Bureau for Statistics. Direct sharing of the data used for this research is therefore impossible. Dutch law allows the use of electronic health records for research purposes under certain conditions. According to this legislation, neither obtaining informed consent from patients nor approval by a medical ethics committee is obligatory for this type of observational study containing no directly identifiable data (Dutch Civil Law, Article 7:458).

## Appendix

See Table 6.

**Table 6:** The theoretical safety mark-up for eight risk groups under five levels of financial reserves

	Safety mark-up in a risk-rated market (€)				
Risk group	0% of average financial reserves	50% of average financial reserves	100% of average financial reserves	150% of average financial reserves	200% of average financial reserves
Entire population	10	11	12	14	16
Individuals aged 0–64	7	8	9	11	13
Individuals aged 65 +	23	25	26	27	29
No morbidity flags	4	6	7	9	11
≥ 1 morbidity flags	26	27	28	30	31
Diabetics (PCG12-15)	34	35	36	38	39
Cluster of conditions (DCG10)	116	117	118	119	120
Extremely costly pharmaceutical usage (PCG38)	2441	2442	2443	2444	2445

The safety mark-up is derived through Eqs 4–7 from the Methods section. For these simulations various levels of financial reserves have been applied, each reflecting a defined percentage of the average financial reserves a Dutch health insurer typically held in 2018–2019 per enrollee (€550) [20, 21]

## Footnotes

1 Coefficient of Variation (CV_x_): $\frac{\sigma }{\mu }$. The coefficient of variation is a measure used to indicate the risk/return trade-off by dividing the standard deviation of the return by the mean (expected) return. A lower value for CV indicates a better risk/return trade-off.

2 (β_x_): $\frac{Covariance({r}_{x},\,{R}_{m})}{Variance({R}_{m})}$. The Beta coefficient is a measure to value the relative risk of an asset versus the overall market. *r*_*x*_ depicts the return on a considered stock, while *R*_*m*_ depicts the return on the overall market. The standard deviation – i.e. the square root of the variance – directly influences the Beta, serving, to some extent, as a proxy for risk.

3 Articles 101 and 104 of the European Solvency II Directive (2009/138/EC) dictate a solvency capital requirement to insurers at a 99.5% confidence level.

4 Articles 144–149 of the Commission Delegated Regulation (EU) 2015/35 supplementing Directive 2009/138/EC define the capital requirement for health insurers subject to a defined variance parameter and the volume of an individual insurer.

5 The health underwriting risk module is one part of the complete Capital Requirements for insurers. Other risk modules are disregarded in this paper for reasons of simplification. As a result, the Capital Requirements discussed in this paper are potentially underestimated.

6 Individuals may, for particular reasons, have cancelled their health insurance at any point during the year (or have enrolled after January 1^st^). In all our analyses, individual-level expenditures are annualized and weighted by the fraction of the year an individual was enrolled. For example, expenditures incurred by individuals that were enrolled for half of the year are doubled and attributed a weight of 0.5 in the analyses. In the subsequent parts of this study, the prevalence of any group is consistently measured in insured years, rather than a total number of contracts.

7 For simplification we add a risk adjuster that contains two classes: ‘consumers that qualify for at least one of the six morbidity adjusters’ and ‘consumers that do not qualify for morbidity adjusters at all’. These could be framed as ‘any pre-existing condition’ or ‘no pre-existing condition’, respectively, in terms of the six morbidity indicators. Our motivation for adding this risk adjuster is that the groups with/without a pre-existing condition (‘healthy’ versus ‘unhealthy’) play an important role in our analysis of heteroscedasticity of residual spending. Inclusion of this risk adjuster ensures that the mean residual spending equals zero for both groups, simplifying the presentation and interpretation of results.

8 V_total,j_= V_incomes,j_+ V_reserves,j_. Moreover, in our estimations we assumed zero financial reserves for insurer *j*, setting V_reserves,j_to €0. These reserves should be amassed eventually. Moreover, Table 6 in the Appendix provides an overview of the effect of various levels of financial reserves on our estimations.

9 As a consequence of this legal structure, the potential enrollment of L versus H could generate additional risk selection incentives in the extraordinary situation where both the mean residual spending and its standard deviation are exactly equal between the two groups with different mean risk equalization payments. Since the SCR is directly influenced by these predicted costs, contracting an individual from the risk group with relatively high mean risk equalization payments results in a larger increase to the amount of capital legally required to be retained, ceteris paribus. In theory, the absence of deviations in predicted residual spending, and the variation thereof, between groups may still be insufficient to abolish risk selection incentives for insurers.

10 Article 1 of the Commission Implementing Regulation (EU) 2015/2013 of 11 November 2015 states that due to the health risk equalization system in place in the Netherlands, ${\delta }_{incomes}$ is set to 2.7% rather than 5% in other Member States. Since risk equalization payments decrease the degree of risk borne by insurers, the ${SCR}_{j}$ is adjusted accordingly.

11 The main point of the notation is that the variation in $SCR$ is mainly determined by ${V}_{total,j}$ and to a smaller extent by ${\delta }_{j}$.

12 The European Insurance and Occupational Pensions Authority (EIOPA) applies a cost of capital rate of 6% for the Solvency II legislation [6].

13 Alternatively, an insurer could build up the required capital by raising overall premiums to enrollees. However, this route is disregarded in this exercise.

14 This form of outlier-risk sharing is actually applied in the German sickness fund insurance [17].

15 Since individuals can quantify for multiple DCGs and more than once for the same class, the mean residual spending for DCGs is different from €0. Moreover, all PCG risk-classes have a mean of €-1.

16 Twin products are nearly-identical basic-insurance products with different prices in combination with different options for supplementary products. In general, the lower-priced twin comes with less generous (supplementary) coverage than the higher-priced twin.

## Code availability

Upon request.
